# Response Surface Methodology for Optimizing the Design Parameters of Ultrasonic Liquid-Level Measurement System

**DOI:** 10.3390/mi16111281

**Published:** 2025-11-13

**Authors:** Wanjia Gao, Wendong Zhang, Yue Tian

**Affiliations:** 1 State Key Laboratory of Extreme Environment Optoelectronic Dynamic Measurement Technology and Instrument, North University of China, Taiyuan 030051, China; wdzhang@nuc.edu.cn; 2 Key Laboratory of Instrumentation Science & Dynamic Measurement, Ministry of Education, North University of China, Taiyuan 030051, China; 3 State Key Laboratory of Dynamic Measurement Technology, North University of China, Taiyuan 030051, China; 20220120@nuc.edu.cn

**Keywords:** ultrasonic-level measurement, response surface method, parameter optimization, Box–Behnken design

## Abstract

This study addresses the high-precision requirements for liquid-level detection of propellants in aerospace rockets and optimizes the design parameters of an ultrasonic liquid-level measurement system based on the response surface method (RSM). Meanwhile, a quantitative correlation model between multiple physical parameters and output voltage is established through theoretical derivation. Firstly, the effects of piezoelectric ceramic sheet diameter, ultrasonic frequency, excitation voltage and liquid temperature on the output voltage are investigated. The optimum conditions were obtained by one-way tests, where the output voltage reached its maximum when the diameter of the piezoelectric ceramic sheet was 15 mm and the frequency was 1 MHz. The excitation voltage was positively correlated with the output voltage. Elevated liquid temperature enhanced the echo amplitude. The influence of law remained consistent across different liquid levels. Subsequently, under the liquid level of 12 cm (half-full operating condition), a three-factor, three-level response surface methodology (RSM) analysis experiment was conducted, focusing on three factors that significantly affect energy transfer efficiency: piezoelectric ceramic sheet diameter (*D*), ultrasonic frequency (*f*), and liquid temperature (*T*). The best parameter combination was obtained through model optimization: *D* = 14.773 mm, *f* = 0.878 MHz, *T* = 33.661 °C. The predicted *U*-value was 8.976 V. The validation experiments demonstrated that the error rates between the measured average voltage values and the predicted values under different liquid levels were all <1%, and the coefficient of variation (CV) of the output signal was reduced to 0.9%. This not only meets the error requirements for aerospace liquid-level measurement but also verifies the reliability of the optimized model. This study significantly enhances the output signal stability and measurement accuracy, providing support for the liquid-level detection of aerospace propellants and high-precision liquid-level measurement in industrial applications.

## 1. Introduction

Liquid-level measurement technology holds significant application value in fields including petrochemical engineering, food and pharmaceutical industries, and water conservancy projects. In the aerospace field, liquid-level detection helps monitor and prevent potential fuel leaks or system failures, thereby ensuring operational safety. Therefore, high-precision liquid-level detection is crucial for guaranteeing mission success, safeguarding the safety of spacecraft and astronauts, and optimizing fuel utilization to the greatest extent [1]. Currently, mainstream detection methods include ultrasonic [2], radar [3], capacitive [4], optical [5], float-type [6], and differential pressure-type [7] techniques. However, due to the need to balance high precision, wide temperature adaptability, and low-cost requirements in this field, existing methods have their respective limitations. Among these, the ultrasonic detection method has emerged as a research focus in the fields of aerospace and industrial measurement and control, owing to its characteristics of non-contact, high precision, and strong adaptability [8]. Among them, the ultrasonic impedance method realizes liquid-level detection by analyzing the reflection characteristics of sound waves at the medium interface, and the measurement accuracy under different system environments directly influences the system performance. Zhang, B. et al. conducted research on liquid-level measurement technology based on the ultrasonic impedance method. They successively proposed an external measurement method based on continuous sound wave amplitude, a sealed container measurement scheme focusing on ultrasonic impedance and the “energy circle” inside metal walls, and a method based on the energy balance of a “one-transmit-two-receive” transducer, all of which achieved effective measurement [9,10,11]. However, existing studies have shown that ultrasonic waves are susceptible to the influence of design parameters during propagation, including piezoelectric material properties [12], excitation frequency [13], and medium temperature [14]. These factors can cause signal attenuation or waveform distortion, thereby affecting measurement reliability. Therefore, investigating the influence of system design parameters on ultrasonic energy transfer efficiency has important theoretical significance and engineering value for optimizing the measurement system.

In terms of the optimization of ultrasonic detection systems, scholars worldwide have carried out extensive research. Scholar Hosseingholilou, B. et al. [15] optimized the operating parameters of an ultrasonic seawater desalination system using the response surface method, achieving the optimal performance with a water production rate of 200.737 mL and a salinity of 545 ppm within 1 h of operation, and demonstrated that its operating cost is lower than that of traditional reverse osmosis and multi-stage flash evaporation methods. Meng, L.H. et al. [16] optimized ultrasonic transducer parameters through finite element simulation and analyzed the optimal combination and the order of influencing factors using range analysis; Xu, C. et al. [17] proposed an ultrasonic transducer frequency tracking method based on echo peaks to optimize the operating frequency of ultrasonic transducers. Piezoelectric components serve as core functional carriers in precision measurement and control systems, and their performance directly determines the overall accuracy of the system. Chen, X. D. et al. [18] designed a monolithic self-sensing precision stage with a smart piezo stack and revealed the stack’s crosstalk characteristics via finite element analysis and experiments to achieve system hysteresis compensation and accuracy improvement. Li, W. et al. [19] proposed a BP neural network-based NARMAX model for online construction of piezoelectric actuator hysteresis models, enabling accurate motion control without pre-establishing dynamic models.

However, the traditional single-factor test method struggles to reveal the interactions between multiple parameters [20]. Response Surface Methodology (RSM), as an advanced experimental design and optimization method, can effectively solve this problem. Proposed by Box and Wilson in 1951, this method has been widely used in process optimization across fields, including chemical engineering and the food industry [21]. In recent years, RSM has also shown unique advantages in the field of ultrasonic detection. Yin, Z.H. et al. [22] optimized the parameters of a nonlinear Lamb wave non-destructive testing system for carbon fiber-reinforced polymer laminates using the response surface method, significantly enhancing the detection accuracy and reliability. Tan, L.C. et al. [23] optimized the key parameters for ultrasonic longitudinal wave detection of aluminum plates using the response surface method. Xu, M.H. et al. [24] optimized the process parameters of ultrasonic-microwave-assisted extraction of juglone from walnut green husks using the response surface method, resulting in an actual juglone content of 836.45 μg/g. Ying, Y.B. et al. [25] optimized the ultrasonic-assisted freezing process parameters for shrimp using the response surface method, reducing the time required to pass through the maximum ice crystal formation zone to 105.5 s, which significantly improved the freezing quality.

This study focuses on the optimization of design parameters in the liquid-level measurement system for room-temperature propellants in aerospace rockets and innovatively introduces RSM to the field of optimizing design parameters for ultrasonic liquid-level measurement systems. First, the influences of the piezoelectric ceramic diameter, ultrasonic frequency, and liquid temperature on the output voltage were analyzed through single-factor experiments; second, the Box–Behnken design (BBD) was employed to conduct three-factor, three-level response surface experiments, and establish a multi-parameter optimization model; finally, the model predictive capability was verified through experiments, providing a new approach to improving measurement accuracy. The research findings not only possess theoretical value but also hold important guiding significance for industrial applications.

## 2. Theory and Methods

### 2.1. Core Selection and Platform Construction of Liquid-Level Measurement System

This study is conducted in the specific context of liquid-level detection for room-temperature propellant storage tanks of launch vehicles in the aerospace sector. Table 1 compares the characteristics of current mainstream methods from multiple dimensions.

As shown in Table 1, the radar method and optical method exhibit high sensitivity, but their cost is 3–5 times that of the ultrasonic method. The float method and capacitive method are low-cost, yet their sensitivity fails to meet the “±1 mm” measurement requirement for propellants. Additionally, these methods are all contact-type measurements and thus unsuitable for closed containers. Due to wave attenuation, the ultrasonic pulse echo method is not applicable to propellants with high bubble content or large containers. In summary, this study selects the ultrasonic impedance method for liquid-level measurement. However, when the temperature exceeds the range of −20~80 °C, the performance degradation of piezoelectric elements results in significant signal fluctuations. Therefore, parameter optimization is required to improve temperature adaptability and signal stability.

In this experiment, PZT-5H piezoelectric ceramic sheets (Zibo Yuhai Electronic Ceramic Co., Ltd., Zibo, China) were used as the core sensitive component of the ultrasonic probe. This type is widely applied in liquid-level measurement scenarios due to its high sensitivity and stability over a wide temperature range, and its parameters can meet the requirements of aerospace measurement environments. Its key performance parameters directly affect the transmission and reception efficiency of ultrasonic signals as well as the output voltage characteristics, with specific parameters listed in Table 2.

An aluminum alloy 2219 (Al alloy 2219) container was used in the experiment, with a wall thickness of 3 mm. As specified in GJB 390A-2008 Specification for Aluminium Alloy Sheets and Plates for Aerospace Applications [26], Al alloy 2219 exhibits high specific strength and superior resistance to hydrogen embrittlement—key properties that make it the primary material for liquid rocket propellant storage tanks. Moreover, the 3 mm wall thickness of the experimental container aligns exactly with the actual wall thickness of secondary propellant storage tanks in small launch vehicles, thereby ensuring a high level of consistency between the experimental setup and the target engineering application.

Two piezoelectric ceramic sheets are mounted on the outer wall of the container, functioning as the transmitter and the receiver, respectively. The sheet is mounted on the container wall using a medical-grade silicone (thickness: 0.5 mm; thermal conductivity: 0.23 W/(m·K)). Two key reasons support this choice: first, the high and low temperature resistance of silicone (operating range: −60~200 °C) enables adaptation to the extreme temperature environment of propellant storage, preventing the failure of the mounting structure caused by sudden temperature changes. Second, the elastic properties of silicone ensure tight contact between the sensor and the container wall. As specified in NB/T 47013.3-2024 Non-Destructive Testing of Pressure Equipment—Part 3: Ultrasonic Testing [27], the acoustic impedance matching degree of silicone (approximately 1.5 Mrayl) is superior to that of traditional butter couplants, which can reduce the reflection loss of acoustic energy at the coupling interface and improve the stability of signal acquisition.

In this study, water was used as the test liquid for experimental research. Water serves as an ‘acoustically equivalent simulation medium’ for room-temperature aerospace propellants, allowing the research findings derived from it to be directly extrapolated. Table 3 presents the acoustic parameters of water and commonly used room-temperature aerospace propellants.

As can be seen from Table 3, the acoustic similarity between water and unsymmetrical dimethylhydrazine (UDMH) and N_2_O_4_ is both ≥92%, and their core ultrasonic propagation characteristics can be completely equivalent. Substituting the acoustic impedance values of the three liquid media into Equation (7), respectively, it is found that the transmissivity differences among the three liquids are <3%, and their acoustic impedances are of the same order of magnitude. Therefore, the research conclusions on water are also applicable to real propellants, and only water is selected as the test liquid in this paper.

**Figure 1:**
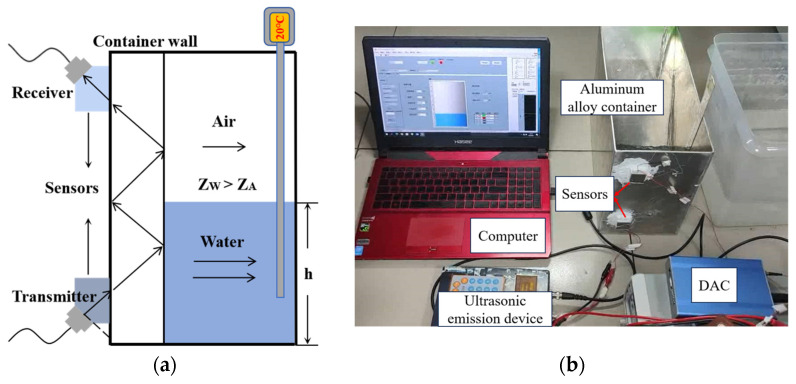
Measurement principle and experimental platform. (**a**) Schematic of liquid-level measurement principle. (**b**) Photograph of the experimental platform.

In this study, the liquid levels measured were 5 cm, 12 cm, and 18 cm, covering the core intervals of low, medium, and high liquid levels. By varying different parameters and simultaneously measuring the results at these three liquid levels, the system error was reduced. The test environment and parameters employed in this study are presented in Table 4, while the equipment used in the experiments, along with their specific models and manufacturers, are listed in Table 5. The schematic diagram is shown in Figure 1a, and the physical construction of the experimental platform is depicted in Figure 1b.

### 2.2. Principle of Liquid-Level Measurement by Ultrasonic Impedance Method

In an ultrasonic liquid-level measurement system, the piezoelectric ceramic sheet serves as the core component, with its electromechanical coupling properties governed by the d-type piezoelectric equation [28]. For transversely isotropic piezoelectric ceramics (polarized along the 3-axis), when considering solely the coupling between the electric field in the 3-direction (the direction of excitation voltage) and mechanical vibration in the 1-direction (the direction of ultrasonic emission), the d-type piezoelectric equation simplifies to the following:

(1) $$T_{1}\;=\;c_{11}^{E}S_{1}\;-\;e_{31}E_{3}$$

(2) $$D_{3}=e_{31}S_{1}+\varepsilon_{33}^{S}E_{3}$$

Among these, *T*_1_ represents the mechanical stress in the 1-direction, Pa, which determines the vibration intensity of the ceramic; *S*_1_ denotes the mechanical strain in the 1-direction (dimensionless), which is related to the thickness of the ceramic sheet; *e*_31_ is the piezoelectric stress constant for the electric field in the 3-direction and stress in the 1-direction, C/m^2^; *E*_3_ stands for the electric field intensity in the 3-direction, V/m; and *D*_3_ refers to the electric displacement vector in the 3-direction, C/m^2^.

In this study, the core indicator of ultrasonic energy transmission efficiency is the ultrasonic emission power *P* of the piezoelectric ceramic, which is directly related to mechanical vibration energy. When internal losses of the ceramic are neglected, the mechanical vibration power can be derived from the stress–strain relationship.

(3) $$w=\frac{1}{2}T_{1}S_{1}$$

Substituting Equation (1) into Equation (3) to eliminate *T*_1_, we obtain

(4) $$w=\frac{1}{2}(c_{11}^{E}S_{1}-e_{31}\cdot \frac{U}{d})S_{1}$$

When the system reaches steady-state vibration, strain *S*_1_ exhibits a linear correlation with the piezoelectric constant and excitation voltage. Meanwhile, by introducing the temperature-dependent mechanical efficiency *η*(*T*), the relationship between the ultrasonic transmission power *P_em_* and the core parameters can be derived as follows:

*P_em_* *∝* |*d*_31_|·*U_ex_*·*S*·*f*·*η*(*T*) (5)

Equation (5) clearly quantifies the correlation logic between ultrasonic emission power and core parameters. *η*(*T*) is the temperature correction term, *η*(*T*) = 0.8 − 0.002(*T* − 20). *d*_31_ is a constant whose absolute value determines the vibration intensity under the same voltage. *U_ex_* (excitation voltage) drives vibration linearly. *S* (radiation area) is determined by the diameter of the piezoelectric sheet, but a balance between radiation and loss must be maintained [29]. *f* is the resonant frequency of the piezoelectric ceramic sheet. Multiple parameters work synergistically; optimizing the key parameters can maximize the ultrasonic energy transfer efficiency, thereby improving the signal intensity and stability of the measurement system.

The ultrasonic transmitting device emits a set of square waves with a duration of 10 μs and is triggered once every 30 ms. The excitation voltage is adjustable from ±5 V to ±15 V. After ultrasonic waves are transmitted into the container, wave reflection, transmission, and refraction occur within the thin wall of the container. The sound intensity reflection coefficient (*R*) and the sound intensity transmission coefficient (*T*) satisfy the following equation [30]:

(6) $$R=\frac{I_{a}}{I}=\frac{{\left(Z_{2}-Z_{i}\right)}^{2}}{{\left(Z_{2}+Z_{i}\right)}^{2}}$$

(7) $$T=\frac{I_{t}}{I}=1-R=\frac{4Z_{2}Z_{i}}{{\left(Z_{2}+Z_{i}\right)}^{2}}$$

Here, *I_a_* denotes the reflected sound intensity, W/m^2^; *I_t_* denotes the transmitted sound intensity, W/m^2^; *I* denotes the incident sound intensity, W/m^2^; *Z*_2_ denotes the acoustic impedance of the tested container, Mrayl; *Z_i_* denotes the acoustic impedance of the internal medium, Mrayl. From Equation (7), it can be observed that the more ultrasonic waves are transmitted into the container, the less reflected echo energy there is, and vice versa.

Based on Equations (6) and (7), combined with the parameters of the experimental system in this study, the influence of key parameters on the output voltage can be quantitatively analyzed. From Table 4, the acoustic impedance of Al alloy is obtained as *Z_AL_* = 31.6 Mrayl, and the acoustic impedance of water at 20 °C is *Z_W_* = 1.48 Mrayl. Substituting these values into Equation (6), the reflectivity *R_W_* is calculated to be approximately 82%, with the transmissivity *T_W_* being 18%. The acoustic impedance of air at 20 °C is *Z_A_* = 4 × 10^−4^ Mrayl. Substituting it into Equation (6) gives a reflectivity *R_A_* of approximately 99.96%. It can thus be concluded that air has a weaker ability to transmit ultrasonic waves than water, and most of the ultrasonic energy is reflected at the air–Al alloy interface [31]. Therefore, when there is a certain height of liquid between the two transducers, it alters the reflection and transmission characteristics of ultrasonic waves, and the residual energy transmitted to the receiver also changes. The liquid levels at different heights inside can be measured according to the transmission efficiency of ultrasonic waves, that is, the voltage value of the residual ultrasonic energy received by the receiver.

When the piezoelectric ceramic diameter increases from 10 mm to 15 mm, the radiation area of the sensor increases from 0.785 cm^2^ to 1.767 cm^2^. The incident sound intensity *I* is positively correlated with the area, increasing by 125%. Derived from Equation (7), the transmitted sound intensity *T* increases to 23%, indicating that the diameter affects the ultrasonic energy transfer efficiency.

At 30 °C, the acoustic impedance of water is *Z_w_* = 1.5 Mrayl. Substituting this value into Equation (7) gives a transmissivity of *T_W_* ≈ 24%, which represents a 4.3% increase compared to that at 20 °C. Thus, the higher the temperature, the higher the ultrasonic transmission efficiency.

### 2.3. Piezoelectric–Ultrasonic Propagation Coupling Modeling

Based on the piezoelectric effect theory and acoustic wave propagation theory, a piezoelectric–ultrasonic propagation coupling mathematical model is established to provide theoretical support and a verification tool for the proposed RSM optimization method. This paper derives the functional relationships between piezoelectric transmission power, ultrasonic propagation loss, and output voltage in modules, and finally obtains a general model, laying a theoretical foundation for subsequent model verification.

#### 2.3.1. Derivation of Ultrasonic Propagation Loss

In the ultrasonic impedance method, the actual propagation path of ultrasonic waves is transmitter–coupling agent–container wall–multiple reflections at the interface between the inner wall of the container and the internal medium–container wall–coupling agent–receiver. The total ultrasonic propagation loss mainly consists of three parts: the transmission loss at the coupling agent–container wall interface (*T_cw_*), the multiple reflection loss at the inner wall–medium interface (*R_c_*), and the medium transmission loss (*A*(*h*,*T*)). Among them, the transmission rate of silica gel (T_cp_) is 0.92; the transmission rate at the 2219 aluminum alloy–water interface (*T_wall_*) can be calculated by Equation (7), which is approximately 17.1%. *R_c_* satisfies Equation (6). The round-trip transmission loss of water is approximately

*T_all_* = (*T_cp_*·*T_wall_*)^2^ (8)

The magnitude of the medium attenuation loss is related to the liquid-level height. Considering the influences of frequency and temperature, the propagation loss factor satisfies Equation (9).

*A*(*h*,*T*) = *exp*(−0.5·*α*_0_(*T*)·*f*^2^·*ξ*(*T*)·*h*) (9)

Among them, *h* is the liquid-level height, which ranges from 5 to 18 cm in this study. *α*_0_(*T*) denotes the reference attenuation coefficient, and *ξ*(*T*) represents the attenuation temperature correction term. Both terms are obtained by fitting the measured data and satisfy Equations (10) and (11).

*α*_0_(*T*) = 0.015*T* + 18.5 (10)

*ξ*(*T*) = 1 − 0.008 (*T* − 20) (11)

At 20 °C and an ultrasonic frequency of 1 MHz, when *h* is 0, 5, 18, and 20 cm, respectively, the values calculated by Equation (9) are as follows: *A*(0,20) = 1, *A*(5,20) = 73.6%, *A*(18,20) = 33.1%, *A*(20,20) = 29.2%. This conclusion is consistent with the theory presented in Section 2.2.

In summary, the ultrasonic propagation loss is defined as the ratio of the received ultrasonic power (*P_rec_*) to the transmitted ultrasonic power (*P_em_*), which satisfies Equation (11).

(12) $$\frac{P_{rec}}{P_{em}}=T_{all}\cdot R_{c}\cdot A(h,T)$$

#### 2.3.2. Derivation of Input Voltage 

The transducer at the receiving end converts ultrasonic energy into voltage, and the relationship between the output voltage *U* and the received power *P_rec_* is *U* ∝ $\sqrt{P_{rec}}$. Combined with the law of conservation of energy, the output voltage is finally derived as follows:

(13) $$U=K\cdot d_{t}(D)\cdot \frac{g\left(f\right)\sqrt{f\cdot \eta (T)}}{\exp\left(0.5\cdot \alpha_{0}\left(T\right)\cdot f^{2}\cdot \xi \left(T\right)\cdot h\right)}$$

Among them, *K* is the comprehensive coefficient, which is obtained by fitting the measured data with a value of *K* = 28.5; *g*(*f*) represents the frequency gain, with the expression *g*(*f*) = exp[−4.0(*f* − 1.0)^2^]. The derived quantitative coupling relationship between the output voltage *U* and piezoelectric ceramic diameter *D*, ultrasonic frequency *f*, liquid temperature *T*, and liquid-level height *h* provides a mathematical basis for subsequent model verification and method advantage analysis.

Based on the aforementioned liquid-level detection principle and experimental platform, different sensors were used to investigate the effects of piezoelectric ceramic sheet diameter, center frequency, excitation voltage amplitude, and liquid temperature on the output voltage. The receiving sensor was connected to a host computer via a digital acquisition card. The received signal waveform was displayed in real time on the host computer, and the signal amplitude was recorded as the output voltage. Different liquid temperatures were measured using a digital probe thermometer.

## 3. Measurement and Discussion

### 3.1. Single-Factor Test of Ultrasonic Liquid-Level Detection

#### 3.1.1. The Influence of the Diameter of the Piezoelectric Ceramic Sheet

**Figure 2:**
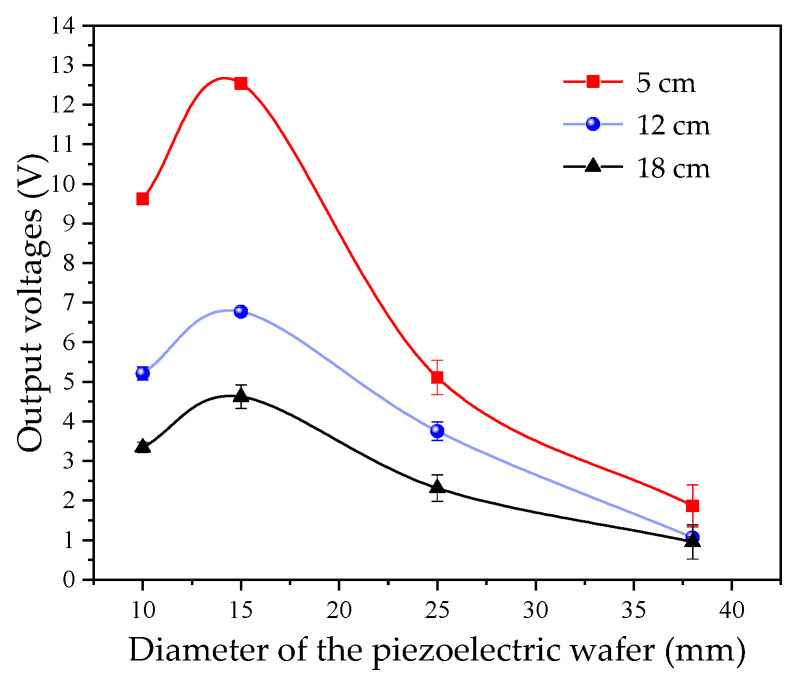
Effect of piezoelectric ceramic sheet diameter on output voltage.

As can be seen from Equation (5), the ultrasonic emission power *P* is related to the radiation area *S*, which is determined by the diameter of the piezoelectric ceramic sheet. The piezoelectric ceramic sheet’s diameter typically ranges from 10 to 30 mm. In this study, piezoelectric ceramic sheets with a center frequency of 1 MHz, the peak-to-peak value of the excitation voltage is ±15 V. Diameters of 10 mm, 15 mm, 25 mm, and 38 mm were tested on the measurement platform shown in Figure 1b, to investigate the effect of different diameters on the detection results. The liquid levels were set to 5 cm, 12 cm, and 18 cm, respectively. For each liquid level, 10 repetitions were conducted for each experimental group, and the average value was calculated. The measurement results are plotted in Figure 2.

As observed in Figure 2, on the experimental platform used in this study, under different liquid levels, the peak values of the output voltage vary; however, their variation trends with diameter are identical, reaching the maximum when the diameter is 15 mm. When the diameter is excessively large, an increase in diameter leads to an expansion of the near-field region of the transmitted wave and a loss of ultrasonic transmission energy. Therefore, the selection of the diameter of the piezoelectric ceramic sheet is crucial in liquid-level detection. The diameter term *d_t_*(*D*) in the output voltage *U* can be obtained by fitting the experimental results, which satisfies Equation (14).

*d_t_*(*D*) = −0.016*D*^2^ + 0.44*D* − 2 (14)

#### 3.1.2. The Influence of Ultrasonic Frequency

**Figure 3:**
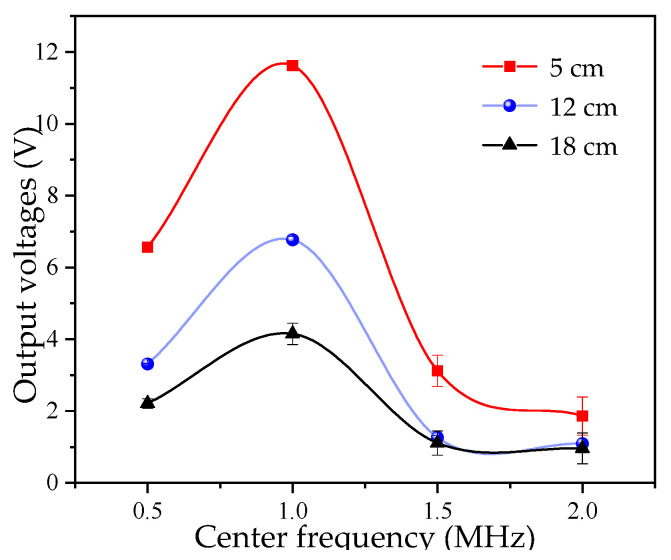
Effect of piezoelectric ceramic sheet center frequency on output voltage.

When an ultrasonic detection instrument is paired with the probe, the probe’s natural frequency is the most important parameter [32]. The frequency used in ultrasonic testing typically ranges from 0.5 MHz to 10 MHz. Therefore, in this study, piezoelectric ceramic sheets with a diameter of 15 mm, the peak-to-peak value of the excitation voltage is ±15 V, and natural frequencies of 500 kHz, 1 MHz, 1.5 MHz, and 2 MHz were tested on the measurement platform shown in Figure 1b to investigate the effect of frequency on the test results. For each liquid level, 10 repetitions were conducted for each experimental group, and the average value was calculated. The measurement results are plotted in Figure 3.

As observed in Figure 3, the test results are most favorable at a frequency of 1 MHz. This is because, as the natural frequency increases, the diffraction angle decreases, and the wave energy becomes more concentrated. However, as the frequency increases, the near-field length increases, and the energy attenuation increases sharply. Thus, the selection of the piezoelectric ceramic sheet’s center frequency is also a key influencing factor.

#### 3.1.3. The Influence of the Excitation Voltage

**Figure 4:**
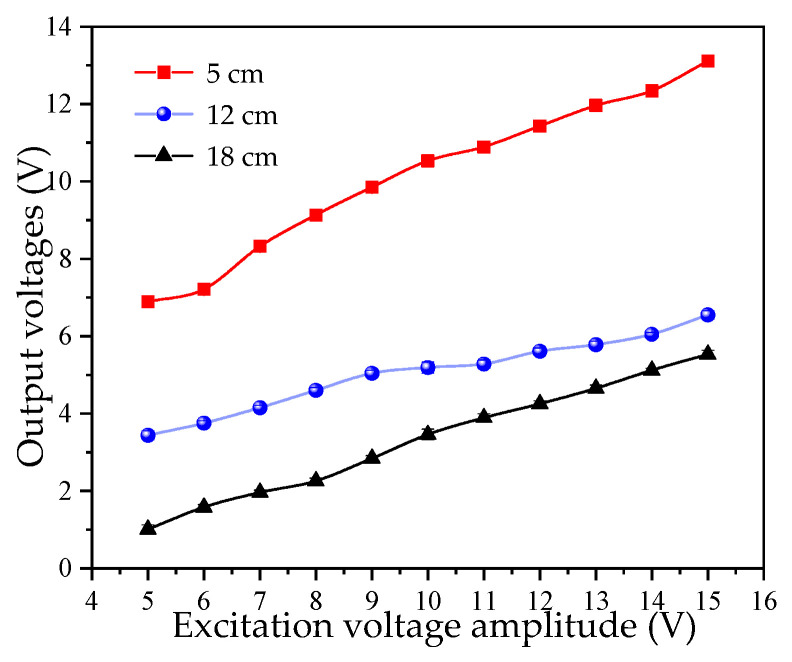
Effect of excitation voltage amplitude on output voltage.

An ultrasonic transducer emits ultrasonic waves and requires a front-end electrical signal to excite the piezoelectric ceramic sheet to vibrate [33]. The liquid-level system in this study is based on the ultrasonic impedance method and detects the liquid level by measuring the ultrasonic energy received at the receiver. The total energy is reflected in the peak voltage of the signal [34]. This study uses a continuous sine wave as an excitation signal. A piezoelectric ceramic sheet with a diameter of 15 mm and a center frequency of 1 MHz was selected for measurement. A sine wave with an excitation frequency of 1 MHz was used, with its peak-to-peak voltage *U_ex_* gradually increasing from ±5 V to ±15 V. Measurement data were recorded at intervals of ±1 V. For each liquid level, 10 repetitions were conducted for each experimental group, and the average value was calculated. The measurement results are plotted in Figure 4.

As observed in Figure 4, the experimental results exhibit a certain degree of linearity. The greater the peak-to-peak value of the excitation signal voltage, the more ultrasonic energy is received. Therefore, in the subsequent research, the excitation voltage *U_ex_* of the ultrasonic wave is fixed at ±15 V. Meanwhile, ±15 V is also the standard power supply voltage.

#### 3.1.4. The Influence of Liquid Temperature

Temperature differences alter the acoustic impedance of the medium, thereby influencing the reliability of the system. In this study, silicone was used for sensor mounting. The thermal conductivity of silicone generally ranges from 0.1 to 0.2 W/(m·K), indicating relatively weak thermal conduction capability—this creates a certain degree of thermal resistance during heat transfer. In our experimental setup, the sensor is connected to the aluminum alloy container wall via silicone; due to the low thermal conductivity and high thermal resistance of silicone, heat transfer between the sensor surface and the liquid proceeds relatively slowly, making it difficult to form a significant temperature difference between the two. Furthermore, in practical application cases, when materials with low thermal conductivity are used for sensor mounting, if the liquid temperature is relatively stable, the difference between the sensor surface and the liquid is typically within a negligible range. In conclusion, since this study focuses primarily on the effect of liquid temperature on ultrasonic energy transfer, additional monitoring of the sensor surface temperature is unnecessary.

**Figure 5:**
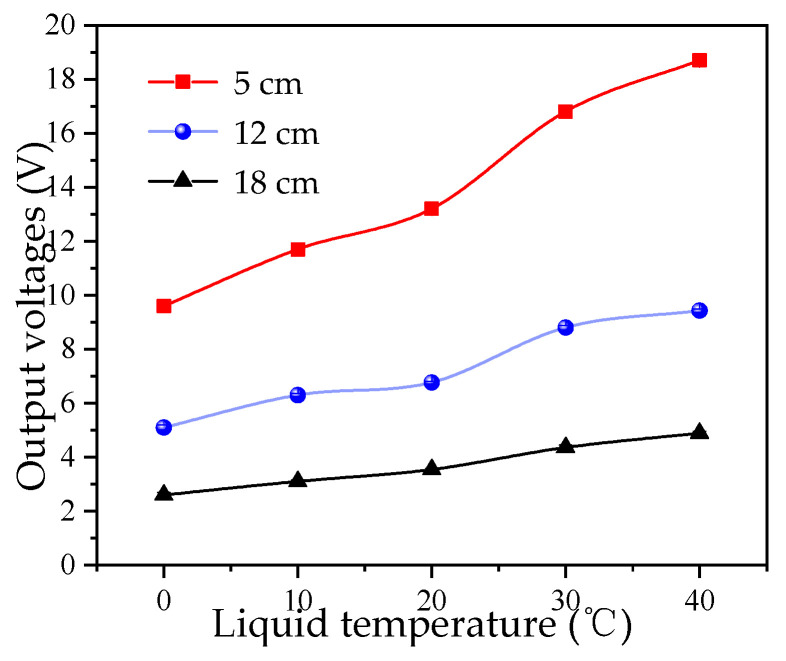
Effect of liquid temperature on output voltage.

The propagation velocity of ultrasonic waves varies with the liquid temperature, which in turn causes variations in the efficiency of ultrasonic energy transmission [35]. This study investigates the effect of different liquid medium temperatures on the measurement results. Measurements were conducted at different liquid temperatures. A digital probe thermometer was immersed in the water to monitor the temperature, with a variation range of less than ±1 °C. The water temperature varied from 0 °C to 40 °C, and for each liquid level, 10 repetitions were conducted for each experimental group, and the average value was calculated. The measurement results are shown in Figure 5.

As observed in Figure 5, the amplitude of the received echo increases with increasing temperature. This is because when the water temperature is below 74 °C, the acoustic impedance decreases with increasing temperature, leading to an increase in sound velocity and thus higher ultrasonic energy transmission efficiency. Thus, environmental temperature is a common influencing factor.

All the above experimental results are consistent with the theoretical derivation, which ensures the reliability of the experiment. From the single-factor experiments, it can be observed that the influence trends of parameters under different liquid levels on *U* are consistent—this ensures the universality and validity of the model established in this study.

### 3.2. Analysis of the Optimal System Parameters for Ultrasonic Liquid-Level Measurement

#### 3.2.1. Experimental Design

Based on the results of the single-factor experiments, the response surface method (RSM) was used to design the experiments. In this study, the Box–Behnken design (BBD) principle was employed, and three factors that significantly affect the ultrasonic energy transmission efficiency in liquid-level measurement experiments were selected: piezoelectric ceramic sheet diameter *D* (*x*_1_), ultrasonic frequency *f* (*x*_2_), and liquid temperature *T* (*x*_3_). A three-factor, three-level response surface analysis experiment was conducted to analyze the relationship between the received ultrasonic energy (i.e., the output voltage, *U*) and the selected factors. The experiment was designed under the working condition of a liquid-level height of 12 cm (half-full condition). The encoding schemes of each response factor and its levels are shown in Table 6.

#### 3.2.2. Output Voltage Response Model Under the Combined Effect of Multiple Parameters

Based on the above parameters, a response surface experiment was designed, with a total of 15 experimental runs. The tested container used in the experiments was an Al alloy bucket with a wall thickness of 3 mm, and the internal liquid medium was water. Each experimental run was repeated 10 times, and the average of the measurement results was recorded as the response value (output voltage, *U*). The experimental results are shown in Table 7.

The output voltage (*U*) results under different parameters were input into the Design-Expert software. After calculation and fitting, the multiple regression equation for *U* was derived as follows:

(15) $$\begin{array}{l} U=8.79-0.7148x_{1}-1.10x_{2}+0.8278x_{3}+0.3325x_{1}x_{2}-0.123x_{1}x_{3}-0.2125x_{2}x_{3}\\ -2.07{x_{1}}^{2}-4.51{x_{2}}^{2}-1.24{x_{3}}^{2} \end{array}$$

The reliability of the output voltage response model was analyzed using Design-Expert 13.0.1.0 64-bit (Stat-Ease, Inc., Minneapolis, MN, USA, Build date: 11 January 2021). The variance and significance of the model were analyzed, and the results are presented in Table 8.

**Figure 6:**
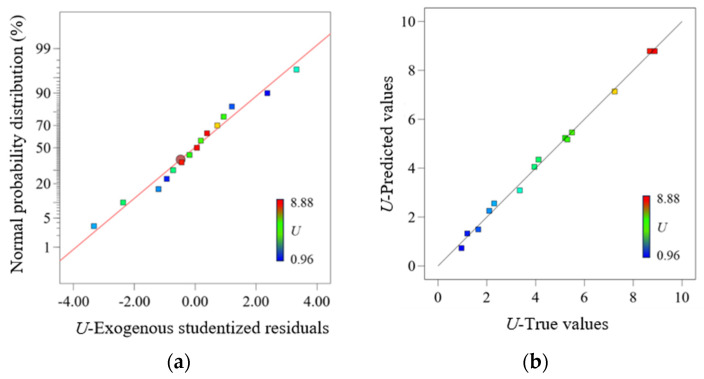
Residual analysis and actual vs. predicted values of output voltage. (**a**) Normal probability plot of residuals. (**b**) Comparison of actual and predicted output voltage values.

In the fitting model of the response surface method, a *p*-value < 0.05 typically indicates a significant model; a *p*-value > 0.1 indicates an insignificant model; and a *p*-value < 0.01 indicates a highly significant model. From the results in Table 8, the model’s F-value is 166, and the *p*-value is <0.0001, indicating a highly significant model. The Lack of Fit F-value is 11.24 with a *p*-value > 0.05, indicating that the model is valid. The determination coefficient R^2^ of the model is 0.9967, which is very close to 1, indicating good predictive capability. The normal probability distribution of the output voltage *U* residuals is presented in Figure 6a, which shows that the model exhibits a strong linear relationship with the normal distribution. The regression model for *U* is relatively accurate, confirming the reliability of the variance analysis and the validity of the regression model. By comparing F-values, the order of influence of the design parameters on *U* is determined as follows: ultrasonic frequency > liquid temperature > piezoelectric ceramic sheet diameter. The *p*-values of all three factors are <0.05, indicating that each factor has a significant effect on output voltage. A comparison of the actual and predicted values of the *U* is presented in Figure 6b.

#### 3.2.3. Response Surface Analysis

**Figure 7:**
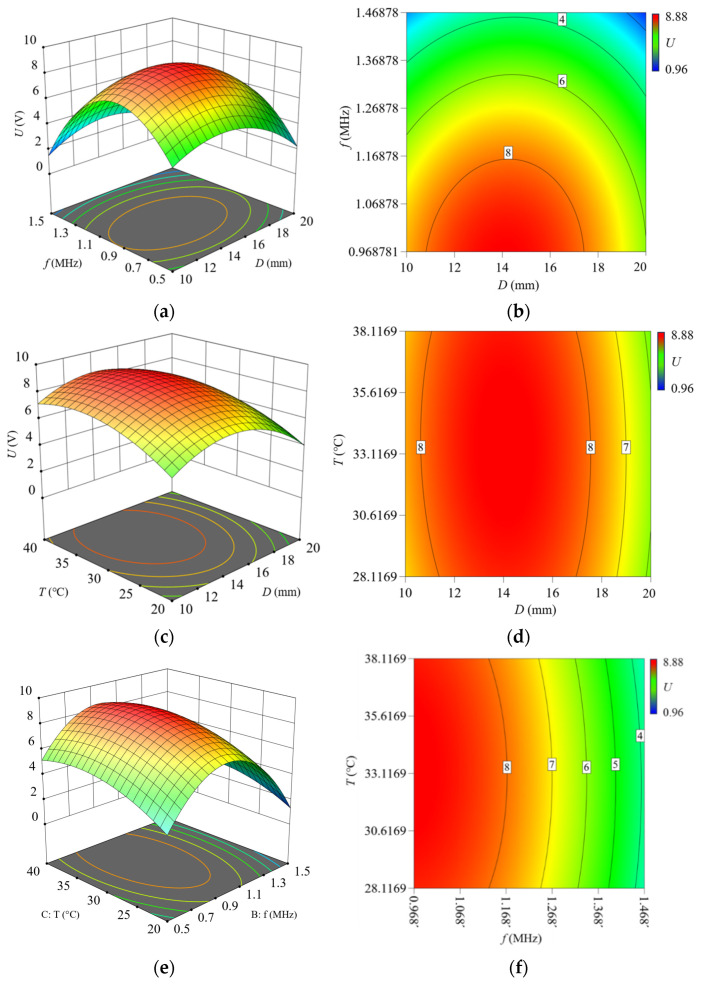
Response surface plots and contour plots showing interactive effects on output voltage. (**a**) Response surface plot: Frequency vs. diameter. (**b**) Contour plot: Frequency vs. diameter. (**c**) Response surface plot: Temperature vs. diameter. (**d**) Contour plot: Temperature vs. diameter. (**e**) Response surface plot: Temperature vs. frequency. (**f**) Contour plot: Temperature vs. frequency.

Based on the model, response surface plots were generated and analyzed to investigate the interactive effects of the piezoelectric ceramic sheet diameter (*x*_1_), ultrasonic frequency *f* (*x*_2_), and liquid temperature *T* (*x*_3_) on *U*. Response surface plots and contour plots of *U* versus key parameters are presented in Figure 7. Figure 7a,b show the response surface plot and contour plot of *U* under the interactive effect of ultrasonic frequency and the diameter of the piezoelectric ceramic sheet. The results show that the output voltage value *U* reaches its maximum within the range of 0.9–1.1 MHz (ultrasonic frequency) and 14–16 mm (diameter).

Figure 7c,d show the response surface plot and contour plot of *U* under the interactive effect of liquid temperature and the diameter of the piezoelectric ceramic sheet. The results show that the output voltage *U* reaches its maximum within the range of 30–35 °C (liquid temperature) and 14–16 mm (diameter).

Figure 7e,f show the response surface plot and contour plot of *U* under the interactive effect of liquid temperature and ultrasonic frequency. The results show that the output voltage *U* reaches its maximum within the range of 30–35 °C (liquid temperature) and 0.9–1.1 MHz (ultrasonic frequency).

#### 3.2.4. Optimization and Verification of System Environment Parameters

In practical liquid-level measurement applications, the more ultrasonic energy received, the more favorable it is for accurate analysis of measurement results. In this study, ultrasonic energy corresponds to the output voltage (*U*) during detection. Therefore, this study explores the optimal combination of piezoelectric ceramic sheet diameter (*D*), ultrasonic frequency (*f*), and liquid temperature (*T*) to achieve a higher *U*. Single-objective optimization of the system design parameters was performed to maximize *U*. During optimization, the boundary ranges for piezoelectric ceramic sheet diameter, ultrasonic frequency, and liquid temperature were consistent with those used in the response surface experiments. The results indicate that the optimal parameter combination for maximizing *U* is piezoelectric ceramic sheet diameter is 14.773 mm, ultrasonic frequency is 0.878 MHz, and liquid temperature is 33.661 °C, with a predicted *U* of 8.976 V.

Liquid-level measurement experiments were conducted using the above optimal parameters. For each liquid-level height (5 cm, 12 cm, and 18 cm), the average *U* from three experiments was taken as the experimental result, and the predicted and experimental results are presented in Table 9. The results show that when the liquid levels are 5 cm, 12 cm, and 18 cm, the relative error rates between the predicted values of *U* and the experimental results are 0.73%, 0.93%, and 0.98%, respectively. All errors are <1%, confirming that the model exhibits good predictive capability for single-objective optimization of liquid-level measurement in this environment and possesses universality. Furthermore, the liquid-level range fully covers the full working conditions as well as the monitoring requirements of aerospace propellant storage tanks.

In the single-factor experiment, the output voltage was 8.6 V when the diameter was 15 mm, and the liquid level was 12 cm. However, 10 consecutive repeated measurements showed that the coefficient of variation (CV) of the output voltage under this parameter was 1.8% (with a standard deviation of 0.155 V). After optimization via the response surface method, the diameter was adjusted to 14.773 mm, the output voltage increased to 8.893 V, the coefficient of variation decreased to 0.9% (with a standard deviation of 0.08 V), and the signal stability improved by 50%.

From the perspective of aerospace application requirements, propellant liquid-level monitoring not only demands high measurement accuracy but also requires low signal fluctuation (to avoid false triggering of the propellant delivery protection mechanism). According to QJ 3262-2005 General Specification for Ultrasonic Liquid-Level Sensors for Aerospace Applications [36], the coefficient of variation (CV) of the sensor output signal must be ≤1.0%. The parameter of 15 mm diameter fails to meet this specification, while the optimized parameter of 14.773 mm fully complies with it. Therefore, this optimization has clear engineering practical value.

To further verify the reliability of the RSM optimization results and the accuracy of the experimental data, based on the output voltage model (Equation (13)) derived in Section 2.3, several key experimental points were selected from Table 7 and Table 9. The theoretical values of the output voltage were calculated and compared with the experimentally measured values; the results are shown in Table 10.

As shown in Table 10, the ultrasonic output voltage prediction model established based on the theoretical derivation in this study exhibits good accuracy, with relative errors generally controlled within 5%. This verifies the scientific validity of the model and the effectiveness of RSM in optimizing environmental parameters, providing reliable theoretical support for the parameter design and performance prediction of ultrasonic testing systems. For the issue of relatively large errors under large-diameter working conditions, the model can be further optimized in subsequent studies by refining the nonlinear relationship between diameter and energy efficiency, thereby expanding its application range.

## 4. Conclusions

Aiming at the problems of insufficient measurement accuracy and poor signal stability in the ultrasonic liquid-level measurement system due to unreasonable matching of design parameters in the liquid propellant-level detection of aerospace rockets, this study optimizes the key design parameters of the ultrasonic liquid-level measurement system using the response surface methodology (RSM). Single-factor experiments under different liquid levels were conducted to determine the influence of piezoelectric ceramic sheet diameter, ultrasonic frequency, excitation voltage, and liquid temperature on the output voltage (*U*), and a quantitative correlation model between multiple parameters and output voltage was established through theoretical derivation, verifying the independent effect of each parameter. The results show that ultrasonic energy transfer efficiency is maximized when the piezoelectric ceramic sheet diameter is 15 mm and the frequency is 1 MHz, while excitation voltage and liquid temperature exhibit a positive correlation with *U*. The influence laws exhibit consistency across different liquid levels. Based on Box–Behnken design (BBD) experiments under the condition of a 12 cm liquid-level (half-full operating condition), three-factor, three-level response surface analysis was performed, focusing on piezoelectric ceramic sheet diameter (*D*), ultrasonic frequency (*f*), and liquid temperature (*T*). A mathematical model relating *U* to the system parameters was developed. The optimization results indicate that when *D* = 14.773 mm, *f* = 0.878 MHz, and *T* = 33.661 °C, the system output voltage *U* reaches a maximum value of 8.976 V, with a verification experiment error of only 0.93%.Under these parameters, experiments were simultaneously conducted at liquid levels of 5 cm and 18 cm, with relative error rates both less than 1%, confirming the reliability and universality of the optimized model. The liquid-level measurement range can cover the full operating condition monitoring requirements of aerospace propellant storage tanks. This study can improve liquid-level measurement accuracy and reduce signal fluctuation, thus possessing the favorable engineering application value.

However, this research has scope for further exploration, including verification of the stability of the optimized parameters, adaptability testing in different liquid media, and evaluation of the anti-interference capability of the system in actual complex environments. Future research could integrate machine learning methods to enhance predictive accuracy and explore multi-objective optimization strategies to balance measurement accuracy and system energy consumption. This research provides a theoretical basis and technical support for the performance optimization of the ultrasonic liquid-level measurement systems and holds an important reference value for industrial applications.

## Figures and Tables

**Table 1:** Comparison of Mainstream Methods for Aerospace Propellant Liquid-Level Measurement.

Method	Sensitivity	Temperature Range	Measurement Range	Response Speed	Disadvantages
Radar method	0.1~0.5 mm	−60~200 °C	0.1~30 m	10~50 ms	High cost, significantly affected by water vapor in the tank.
Capacitive method	1~2 mm	−20~80 °C	0.01~5 m	20~50 ms	Contact measurement, not suitable for closed containers.
Optical method	0.01~0.1 mm	−10~60 °C	0.05~3 m	5~20 ms	Not suitable for opaque propellants and high-temperature scenarios.
Float method	2~5 mm	−40~150 °C	0.1~20 m	100~200 ms	Contact measurement, not suitable for closed containers.
Differential pressure method	1~3 mm	−50~150 °C	0.1~50 m	50~100 ms	Not suitable for low-density and volatile propellants
Ultrasonic pulse echo method	0.5~1 mm	−40~80 °C	0.1~10 m	50~100 ms	Not suitable for high-bubble propellants and large containers.
**Ultrasonic impedance method (this study)**	**0.3~0.8 mm**	**−20~120** °C	**0.05~8 m**	**30~80 ms**	**Parameter optimization is required to improve temperature adaptability and signal stability.**

**Table 2:** Key Parameters of PZT-5H Piezoelectric Ceramics.

Parameter Name	Symbol	Unit	Typical Value
Longitudinal Piezoelectric Strain Coefficient	*d*_33_	m/V	620 × 10^−12^
Transverse Piezoelectric Strain Coefficient	*d*_31_	m/V	275 × 10^−12^
Transverse Piezoelectric Stress Coefficient	*e*_31_	C/m^2^	15.3
Elastic Stiffness Coefficient in Direction 1	$c_{11}^{E}$	Pa	5.56 × 10^10^
Elastic Stiffness Coefficient in Direction 3	$c_{33}^{E}$	Pa	1.17 × 10^11^
Dielectric Constant in Direction 3	$\varepsilon_{33}^{S}$	F/m	2.83 × 10^−8^
Dielectric Loss Factor	*tanδ*	%	≤2.0
Curie Temperature	*T_c_*	°C	230
Mechanical Quality Factor	*Q_m_*	-	70
Disc Diameter	*D*	mm	10–20
Operating Frequency	*f*	MHz	0.5–2.0

**Table 3:** Comparison of Acoustic Parameters Between Water and Room-Temperature Propellants.

Medium Type	Acoustic Impedance (20 °C, Mrayl)	Sound Velocity Temperature Coefficient (m/(s·°C))	Sound Attenuation Coefficient (1 MHz, 20 °C, Np/m)	Acoustic Similarity to Water (%) *	Transmissivity (*T_W_*)
Deionized water (this study)	1.48	2.1	0.003	100	23%
UDMH (tactical missile propellant)	1.32	1.9	0.004	92	21%
N_2_O_4_ (upper-stage launch vehicle propellant)	1.56	2.3	0.0028	95	24%

* The acoustic similarity = (1 − |water parameter − propellant parameter|/water parameter) × 100%, calculated by comprehensively integrating acoustic impedance, sound velocity, and temperature coefficient. A value ≥ 90% indicates ‘acoustic equivalence’.

**Table 4:** Test Environment and Parameters.

Parameter	Symbol	Parameter Value
Container Material	*M_c_*	Al Alloy 2219
Container Wall Thickness	*δ*	3 mm
Probe Material	*M_TX_*	PZT-5H
Liquid-Level Height	*h*	5 cm, 12 cm, 18 cm
Test Liquid	*M_L_*	Water
Coupling Material	*M_cp_*	Organic Silicone
Acoustic Impedance of Al Alloy	*Z_AL_*	31.6 Mrayl
Acoustic Impedance of Water	*Z_W_*	1.48 Mray (20 °C) 1.5 Mrayl (30 °C)
Acoustic Impedance of Air (20 °C)	*Z_A_*	4 × 10^−4^ Mrayl
Temperature Range	*T*	0–40 °C

**Table 5:** Models and Manufacturers of Experimental Equipment.

Equipment	Model	Manufacturer
Digital probe thermometer	TP101	Wenzhou Shengce Instrument Co., Ltd. (Wenzhou, China)
Digital acquisition card (DAC)	USB-1610	Beijing Xinchao Technology Co., Ltd. (Beijing, China)
Ultrasonic transmitting device	ULT-01	North University of China (Taiyuan, China)

**Table 6:** Factor and level for response surface analysis.

Factor	Level		
	Low Value (−1)	Center Point (0)	High Value (+1)
*x*_1_: *D* (mm)	10	15	20
*x*_2_: *f* (MHz)	0.5	1	1.5
*x*_3_: *T* (°C)	20	30	40

**Table 7:** Experimental results of the output voltage.

Run No.	*x*_1_: *D* (mm)	*x*_2_: *f* (MHz)	*x*_3_: *T* (°C)	*U* (V)
1	10	0.5	30	4.12
2	20	0.5	30	2.1
3	10	1.5	30	1.65
4	20	1.5	30	0.96
5	10	1	20	5.21
6	20	1	20	3.95
7	10	1	40	7.24
8	20	1	40	5.49
9	15	0.5	20	3.35
10	15	1.5	20	1.2
11	15	0.5	40	5.3
12	15	1.5	40	2.3
13	15	1	30	8.8
14	15	1	30	8.68
15	15	1	30	8.88

**Table 8:** Analysis of variance (ANOVA) for the output voltage response model.

Source	Sum of Squares (SS)	Degree of Freedom (df)	Mean Square (MS)	F-Value	*p*-Value
Model	108.13	9	12.01	166.00	<0.0001
*x*_1_	4.09	1	4.09	56.47	0.0007
*x*_2_	9.59	1	9.59	132.53	<0.0001
*x*_3_	5.48	1	5.48	75.73	0.0003
*x*_1_ *x*_2_	0.4422	1	0.4422	6.11	0.0564
*x*_1_ *x*_3_	0.0605	1	0.0605	0.8361	0.4024
*x*_2_ *x*_3_	0.1806	1	0.1806	2.50	0.1750
*X*_1_^2^	15.86	1	15.86	219.09	<0.0001
*X*_2_^2^	75.00	1	75.00	1036.21	<0.0001
*X*_3_^2^	5.70	1	5.70	78.74	0.0003
Residual	0.3619	5	0.0724		
Lack of Fit	0.3416	3	0.1139	11.24	0.0828
Pure Error	0.0203	2	0.0101		
Total	108.13	9			
Model Summary Statistics		*R*^2^ = 0.9967		*R*^2^_adj_ = 0.9907	

**Table 9:** Predicted vs. experimental results for single-objective optimization.

Liquid Levels (cm)	Predicted Value (V)	Experimental Value (V)	Error (V)	Relative Error (%)
5	16.675	16.553	0.122	0.73%
12	8.976	8.893	0.083	0.93%
18	6.346	6.408	0.062	0.98%

**Table 10:** Comparison of Output Voltage: Calculated vs. Experimental Values.

Parameter Combination (*D*/mm, *f*/MHz, *T*/°C, *h*/cm)	Calculated Output Voltage *U_calc_*/V	Experimental Measured Output Voltage *U_exp_*/V	Relative Error (%)
15, 1.0, 30, 12	8.84	8.8	0.45
10, 0.5, 30, 12	4.03	4.12	2.18
20, 1, 20, 12	3.3	3.95	16.46
14.773, 0.878, 33.661, 18	6.72	6.408	4.87

## Data Availability

The original contributions presented in this study are included in the article. Further inquiries can be directed to the corresponding author.
